# The Homeodomain-Leucine Zipper Genes Family Regulates the Jinggangmycin Mediated Immune Response of *Oryza sativa* to *Nilaparvata lugens*, and *Laodelphax striatellus*

**DOI:** 10.3390/bioengineering9080398

**Published:** 2022-08-17

**Authors:** Sheraz Ahmad, Yu Chen, Amir Zaman Shah, Huaiqi Wang, Chuanyuan Xi, Haowen Zhu, Linquan Ge

**Affiliations:** College of Horticulture and Plant Protection, Yangzhou University, Yangzhou 225009, China

**Keywords:** homeodomain-leucine zipper, transcriptomic analysis, brown planthopper, small brown planthopper, jinggangmycin

## Abstract

The homeodomain-leucine zipper (HDZIP) is an important transcription factor family, instrumental not only in growth but in finetuning plant responses to environmental adversaries. Despite the plethora of literature available, the role of HDZIP genes under chewing and sucking insects remains elusive. Herein, we identified 40 *OsHDZIP* genes from the rice genome database. The evolutionary relationship, gene structure, conserved motifs, and chemical properties highlight the key aspects of *OsHDZIP* genes in rice. The *OsHDZIP* family is divided into a further four subfamilies (i.e., HDZIP I, HDZIP II, HDZIP III, and HDZIP IV). Moreover, the protein–protein interaction and Gene Ontology (GO) analysis showed that *OsHDZIP* genes regulate plant growth and response to various environmental stimuli. Various microRNA (miRNA) families targeted HDZIP III subfamily genes. The microarray data analysis showed that *OsHDZIP* was expressed in almost all tested tissues. Additionally, the differential expression patterns of the *OsHDZIP* genes were found under salinity stress and hormonal treatments, whereas under brown planthopper (BPH), striped stem borer (SSB), and rice leaf folder (RLF), only *OsHDZIP3*, *OsHDZIP4*, *OsHDZIP40*, *OsHDZIP10*, and *OsHDZIP20* displayed expression. The qRT-PCR analysis further validated the expression of *OsHDZIP20*, *OsHDZIP40*, and *OsHDZIP10* under BPH, small brown planthopper (SBPH) infestations, and jinggangmycin (JGM) spraying applications. Our results provide detailed knowledge of the *OsHDZIP* gene family resistance in rice plants and will facilitate the development of stress-resilient cultivars, particularly against chewing and sucking insect pests.

## 1. Introduction

The static nature of plants entails the frequent endurance of various environmental stresses. In response, plants have developed various mechanisms to adjust to the constantly changing environment [1]. These developmental processes are frequently regulated by numerous transcription factors (TFs) [2]. TFs spreads throughout the genome and can bond with certain functional *c*is-elements, facilitating the plant’s response to various environmental stimuli. The homeodomain–leucine zipper (HDZIP) is a transcription factor family that plays a vital role in plant growth, developmental processes, and stress response [3]. The HDZIP is a class of homeobox proteins containing the homeodomain (HD) and leucine zipper (LZ) motifs [3,4]. These two motifs are the signatures of the HDZIP family and have been found in all eukaryotic species. However, their interaction with a single protein is only found in plants and, therefore, HDZIP in *Plantae* is different than in other organisms [5]. Based on their structure, sequence composition, functional characteristics, and phylogenetic relationship, the HDZIPs are divided into four subfamilies (i.e., HDZIP I, HDZIP II, HDZIP III, and HDZIP IV). Additionally, each subfamily has a unique function and forms a complex interactive network throughout the plant’s developmental phases [6,7,8]. 

The HDZIP subfamily I was found to be highly responsive in the developmental stages and regulates host plant resistance to several abiotic stresses [9,10] and biotic stresses such as pathogens and chilling injury [11]. Previously, the vital role of subfamily II members was reported to be a very light responsiveness to regulating the shade avoidance response and participating in auxin signaling and leaf polarity [12,13,14]. The transcription factors of subfamily III have an obvious effect on plant development through the transportation of various hormones, such as meristem initiation, the formation of microtubule tissues, vascular system development, and the differentiation of apical provinces [15], whereas the subfamily IV members promote the differentiation of epidermal cells in various plant organs, trichome and anthocyanins formation, and attributes in fruit postharvest to abiotic stresses [8,16,17].

In the recent decades, rice consumption has increased globally. A statistical analysis of rice consumption showed a high increase: in the 2008/2009 crop year, 437.18 million metric tons (MMTs) of rice was consumed; in comparison, in 2021/2022, 509.87 MMTs of rice was consumed worldwide. [18]. However, rice crops are targeted by many insect pests in, which the most prominent, BPH *Nilaparvata lugens* Stål and *Laodelphax striatellus* (Hemiptera: Delphacide), are recognized as key pests [19,20]. Both BPH and SBPH directly affect rice through infestations and, in the process, transmit numerous viral pathogens (RGSV) [21,22,23]. Host plant resistance is the most efficient strategy for controlling BPH; however, pesticide preferences are on the top of the list because of their easy availability and prompt results [24]. Furthermore, the recently developed fungicide, jinggangmycin (JGM), is usually applied 2–3 times in rice fields to control rice sheath blight disease (*Rhizoctonia solani*) [25]. However, the JGM is reported to induce rice physiology and biochemistry leading to enhanced BPH flight capacity, body weight, thermotolerance, protein and lipids contents, and fecundity [26,27]. 

Previously, the plant hormone jasmonic acid (JA) is reported to play the main role in host plant defense against numerous herbivores. Herbivore damage elicits a rapid and transient JA burst in the wounded leaves, and JA functions as a signal to mediate the accumulation of various secondary metabolites that confer resistance to herbivores [28]. Brassinosteroids (BRs) are steroid hormones essential for plant growth and development. These hormones control the division, elongation, and differentiation of various cell types throughout the entire plant life cycle. Over the past few decades, studies on BRs caught the attention of plant scientists due to their versatile ability to mitigate various environmental stresses. Additionally, BRs is also involved in maintaining the quality of postharvest produces by enhancing their resistance to abiotic and biotic stress [29]. However, the research is missing highlighting the potential role of these crucial hormones under BPH and SBPH infestations and JGM applications.

Considering the potential role of the *HDZIP* gene family associated with growth and development, physiological and various defense responses, and the diversity of *HDZIP* gene family members in many plant species, it is critical to investigate the global status and evolution of the *HDZIP* gene family in rice [27]. Herein, we, for the first time, investigated the response of the *OsHDZIP* gene family against BPH and SBPH infestations. Additionally, an expression analysis was also performed in rice plants treated with JGM fungicide. Comprehensive in silico analyses were also performed by exploring the rice genome and expression databases. We believe our results will facilitate future research work in the field of rice stress biology, particularly in response to BPH. 

## 2. Materials and Methods 

### 2.1. Investigation of the HDZIP Gene Family Members and Sequence Analysis in Oryza sativa

To obtain insightful knowledge of the *OsHDZIP* gene family regarding sequence analysis in *Oryza* sativa and their counterparts, we retrieved the OsHDZIP proteins from the *Arabidopsis thaliana* genome “TAIR” (https://www.arabidopsis.org/) (accessed on 18 February 2022) [30], the *Cucumis sativus* genome (http://cucurbitgenomics.org/) (accessed on 20 February 2022) [31], and the rice genome database (http://rapdb.dna.affrc.go.jp/) (accessed on 20 February 2022) [32]. To avoid the possible loss of a HDZIP protein due to the fact of missing domain, a local BLASTP with a 1E-5 cutoff was performed. The Conserved Domain Database (CDD) of the National Center for Biotechnology Information (NCBI) (http://www.ncbi.nlm.nih.gov/cdd/) (accessed on 20 February 2022), SMART database (http://smart.embl-heidelberg.de/) (accessed on 18 February 2022) [33], Inter Pro Scan program (https://www.ebi.ac.uk/interpro/) (accessed on 21 February 2022), and Scan Prosite (https://prosite.expasy.org/scanprosite/) (accessed on 18 February 2022) were used to confirm the HDZIP family-specific leucine-zipper (LZ) domain. Additionally, the physiochemical properties of the HDZIP proteins in the studied plants were discovered using the ExPASy online server (http://web.expasy.org/protparam/) (accessed on 21 February 2022) [34].

### 2.2. Phylogenetic Tree, Motif, and Digital Expression Analysis

To obtain detailed knowledge regarding the evolutionary relationship of the HDZIP gene family in rice with the developed genome species. Here, we investigated the phylogenetic relationships of the HDZIP gene family members of rice with model plants. Firstly, the HDZIP amino acid sequences were downloaded from their corresponding genome database and were then aligned through ClustalW software (version 2.1) (http://www.genome.jp/tools/clustalw/) (accessed on 22 February 2022) following the default parameters to examine the evolutionary relationships among the sequences and construct the maximum likelihood phylogenetic tree using MEGA (version 7.0) [35]. Furthermore, the conserved protein motifs of the HDZIP family of *O. sativa* were predicted using the MEME online server (Version 4.12.0) (http://meme-suite.org/) (accessed 10 March 2022) with the default settings. The details of the top 10 predicted motifs were obtained from the MEME suite. The conserved domains of the HDZIP gene family of *O. sativa* were predicted using the NCBI-CDD (http://www.ncbi.nlm.nih.gov/Structure/cdd/wrpsb.cgi) (accessed 10 March 2022). The conserved domain and motif distribution were drawn via Microsoft PowerPoint 365 software. Finally, for the visualization of the heatmap, TBtools (Version 1.098765) was used, and the transcriptomic data were retrieved from the rice genome database and GEO dataset platform from the NCBI [27,32].

### 2.3. Cis-Elements and Gene Ontology of the OsHDZIP Genes

To determine the cis-regulatory elements in each *OsHDZIP* gene, 1.5 kb of an upstream genomic DNA sequence with the starting codon (ATG) was obtained from the rice genome sequence database. Further, we used the plantCARE database (http://bioinformatics.psb.ugent.be/webtools/plantcare/html/) (accessed on 26 February 2022) to identify the cis-elements in the promoter regions of 40 *OsHDZIP* genes of *O. sativa*. Furthermore, for the GO analysis, the OsHDZIP protein sequences were downloaded from the iTAK-Plant Transcription Factor and Protein Kinase Identifier and Classifier (http://itak.feilab.net/cgi-bin/itak/index.cgi) (accessed on 5 March 2022). The obtained OsHDZIP protein sequences were implied in the “CELLO2GO” online server to determine the predicted functions, such as molecular functions, biological processes, and cellular components, and finally, the GO classifications were recovered using Microsoft Excel 365 software.

### 2.4. Interactive Protein Analysis of the OsHDZIP Genes

The online server String (https://string-db.org) (accessed on 8 February 2022) was used for the interactive protein network analysis (accessed on 8 March 2022), using the *O. sativa* OsHDZIP2 protein as a reference following the default advanced settings [36]. Furthermore, pathway enrichment analysis was carried out by searching for OsHDZIP genes in the rice genome database’s online pathway enrichment tool [32].

### 2.5. Prediction of Putative MicroRNAs Targeting OsHDZIP Genes

To predict putative miRNA target sites in the *OsHDZIP* genes in the rice plants under BPH and SBP infestations and JGM spraying applications (briefly described in Section 2), the sequences of rice miRNAs were downloaded from the rice genome database. Moreover, the OsHDZIP CDS sequences were submitted to the online psRNA Target (Server18) with the default parameters for predicting potential miRNAs in *OsHDZIP* genes. The interactive network between the predicted miRNAs and *OsHDZIP* targeted genes were constructed and visualized using Cytoscape software (version 3.919) by the Institute for Systems Biology (Seattle, Washington 98103, USA) following the same procedure by Rizwan et al. [37].

### 2.6. Insect Rearing and Chemical and Stress Treatment

The insects (i.e., BPH and SBPH) and the rice variety used in the study, Ninjing4, were initially obtained from the China National Rice Research Institute insect repository (Hangzhou, China). The Ninjing4 rice variety, known to be resistanceless to these insect infestations, was used for insect rearing. Initially, the BPH and SBPH colonies were reared on the rice seedlings in cement tanks covered with fine mesh outdoors (i.e., natural conditions) for six months (i.e., April to October) and overwintered in lab-controlled conditions. First, we soaked the seeds for 24 h in a water dip plastic tray with a standard size of one-quarter (60 cm H_100 cm W_200 cm L) in standard conditions of 26 ± 2 °C with the 16 h L:8 h D in relative humidity of 80 ± 10% in the ecological laboratory of Yangzhou University. The germinated seeds were transferred to cement tanks covered with fine mesh in an outdoor natural environment and were grown until the six-leaf seedling stage. Secondly, the seedlings were then transferred into plastic pots (dimensions = R ¼ 16 cm). Finally, the stress treatments proceeded at the tillering stage (40 ± 2, 40 ± 4, and 40 ± 8 days).

The JGM technical grade of 61.7% used in this study was obtained from the Qianjiang Biochemistry Co., Ltd. (Haining, Zhejiang, China). Following the protocol of in a previous report by Ahmad et al. (2022), the two hundred parts per million (PPM) solution was Tween 20 obtained from the Sinopsin Group Chemical Reagent Company (Shanghai, China). The fungicide was then sprayed on the rice seedlings, following the procedure in a previous study [27,38].

### 2.7. Expression Profiling of HDZIP Genes in Oryza sativa

Forty day-old (40 ± 2) rice plants were exposed to BPH and SBPH stress, and samples were collected at 2, 4, and 8 days after infestation. Similarly, JGM was sprayed on the rice plants, and samples were taken 2, 4, and 8 days after treatment. The samples were then stored at −80 °C degrees until further experiments. After that, the total RNA was extracted from the samples using kits (Vazyme, Nanjing, China). First, the DNA was removed using DNase I, the concentration and purity were measured with a NanoDrop 1000 spectrophotometer (Thermo Fisher Scientific, Rockford, IL, USA), and the integrity was checked using 1.5% agarose gel electrophoresis. Finally, the resulting cDNA was used as a template for qPCR (quantitative real-time PCR) analysis using SYBR Green real-time PCR master mix (Vazyme, Nanjing, China). The qPCR assays were performed in triplicate using a real-time PCR system (Bio-Rad, Hercules, CA, USA) following the manufacturer’s protocol [27]. 

Furthermore, the 2 μL aliquots of cDNA were amplified by qPCR in 20 μL reaction volumes using the SYBR Premix Ex Taq^TM^ II (TaKaRa, Dalian, China). The cDNAs were amplified at 95 °C for 2 min, followed by 35 cycles of 10 s at 95 °C, then for 30 s, and at 72 °C for 30 s, with a final extension step of 72 °C for 10 min in a CFX96 real-time PCR system (Bio-Rad Co., Ltd., CA, USA). The mRNA amounts of all genes were separately quantified with the stable expression of the constitutive reference gene, actin. The specific primers are listed in (Table S1). After amplification, the target gene cycle threshold (Ct) values were normalized to the reference gene by the 2^−ΔΔCT^ method [39]. The data’s mean values of three biologically independent replicates were used for the final graphs [27].

### 2.8. Statistical Analysis

The data presented in this paper were analyzed using the SPSS software (version 25.0, SPSS Inc., Chicago, IL, USA) for statistical analysis (ANOVA), statistical significance, and a 95% confidence interval (*p* ≤ 0.05). The data were analyzed and are expressed as the mean ± standard deviation (SD) of three biologically independent replicates in all measured parameters, and finally, GraphPad Prism (Version 8.0.2) (GraphPad Software, Inc., LA Jolla, CA, USA) was used for graphical representation [27]. 

## 3. Results

### 3.1. Identification and Sequence Analysis of OsHDZIP Genes in Oryza sativa

We retrieved 40 rice *OsHDZIP* transcription factors from the rice genome database. All genes had a nomenclature of *OsHDZ1* to *OsHDZ40* (Table 1). Among the 40 *OsHDZIP* genes family members, 35 *OsHDZIP* genes resided in the nucleus, whereas *OsHDZIP3*, *OsHDZIP7, OsHDZIP13,* and *OsHDZIP40* were in the plasma membrane, while 2 genes, *OsHDZIP28* and *OsHDZIP37*, resided in the cytoplasm. Other features of *Oryza sativa* OsHDZIP protein were identified, such as locus ID, subfamilies, chromosomal coordinates, molecular weight, chemical properties, and isoelectric point (PI), and tabulated. 

### 3.2. The OsHDZIP Genes Conservative Domain Analysis 

**Figure 1:**
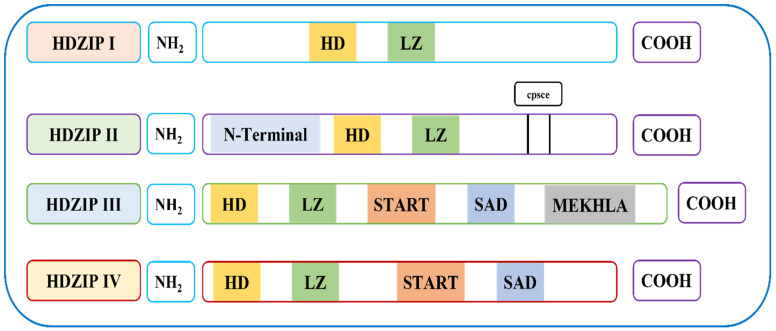
Represents the conserved domain of the *OsHDZIP* genes in *Oryza sativa*.

The HDZIP gene family consisted of two functional domains: homeodomain (HD) and leucine zipper (LZ). Based on their sequence conservation and functional properties, the HDZIP gene family was divided into four subfamilies (i.e., HDZIP I, HDZIP II, HDZIP III, and HDZIP IV). The HDZIP I subfamily contains the HD and LZ domains among the four subfamilies; HDZIP II contains a similar HD and LZ domain with an additional CPSCE motif; HDZIP III and HDZIP IV contain HD and LZ with an additional START and SAD domain. Only HDZIP III possesses the highly conserved MEKHLA domain (Figure 1). The domain distribution and structure of the *OsHDZIP* genes in *Oryza sativa* are shown in Table S3.

### 3.3. Evolutionary Relationship of Oryza sativa HDZIP Genes 

**Figure 2:**
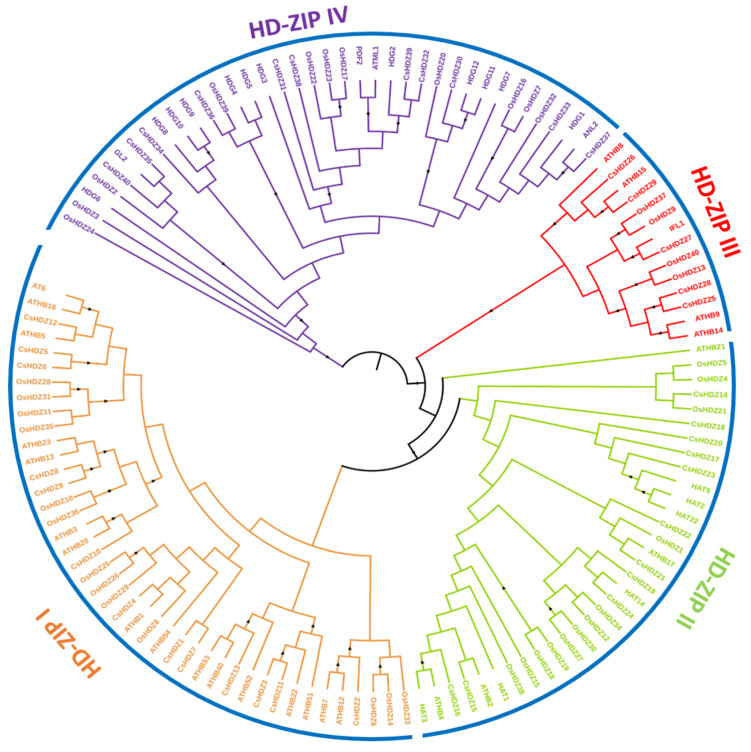
Phylogenetic analysis of HDZIP: the phylogenetic tree was generated using the amino-acid sequences of selected HDZIPs via the maximum likelihood tree method. All *Oryza sativa* HDZIPs, *Arabidopsis thaliana,* and *Cucumis sativus*, with their counterparts, were classified into four subfamilies, and the final tree was displayed using the Interactive Tree Of Life (iTOL) (version 5).

After alignment, a maximum likelihood phylogenetic tree was constructed to gain insightful knowledge regarding the evolutionary relationship of the homeodomain-leucine zipper in *Arabidopsis thaliana* [30], *Cucumis sativus* [8], and *O. sativa* [32] (Figure 2). The protein sequence of the rice (40 OsHDZIP), *A. thaliana* (47 AtHDZIP) [40], and cucumber (40 CsHDZIP) [8] were downloaded from their respective databases and through the maximum likelihood method, the phylogenetic tree was constructed. Following the same procedure as our previously published article, first, all sequences were aligned using ClustalX software with default parameters, then the phylogenetic tree was constructed using MEGA6 software, and the final tree was programmed using Interactive Tree Of Life (iTOL) (version 5) (accessed on 20 March 2022) [41]. Additionally, the OsHDZIP proteins were clustered into four subfamilies based on their phylogenetic relationships: HDZIPI, HDZIPII, HDZIPIII, and HDZIPIV, and the number of OsHDZIP proteins was measured in each subfamily. Subfamily I accounted for 13 proteins, followed by subgroup II with 12 proteins, subfamily IV with 11 proteins, and subfamily III with the least number proteins at 4. 

### 3.4. An Interactive Network of OsHDZIP Protein

**Figure 3:**
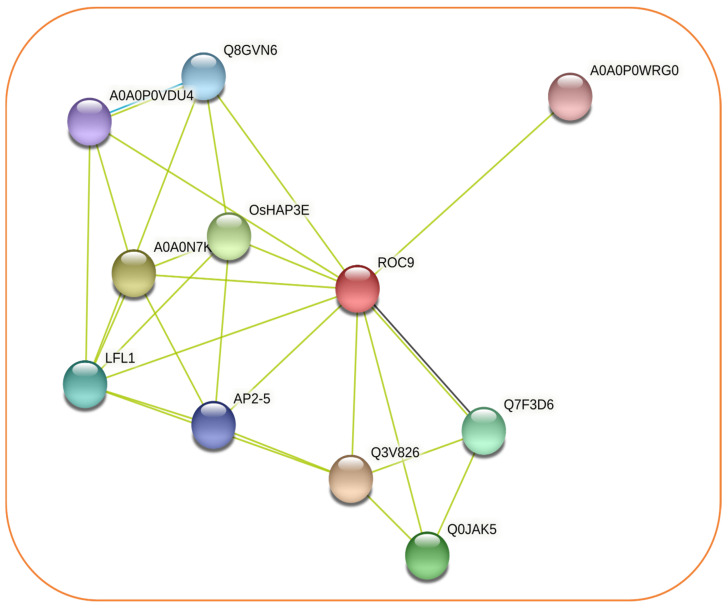
Represents the schematic network of the interactive proteins of the OsHDZIP2 protein of *Oryza sativa*.

The protein interaction analysis revealed various other proteins interacting with the OsHDZIP orthologous gene OsHDZIP2 (Figure 3). The OsHDZIP2 protein from the homeodomain-leucine zipper gene family plays a crucial role in plant growth and stress response. For instance, the YUCCA pathway is the most important and well-characterized pathway that plants deploy to produce auxin, which is the essential hormone in plant development and stress response [42]. In addition, our reference protein, OsHDZIP2, was found to be highly interactive with rice LAZY1 proteins and had an essential role in auxin biosynthesis, the predicted functional partners of OsHDZIP proteins (Table S4).

### 3.5. Prediction of the Potential MicroRNAs Targeting OsHDZIP Genes 

**Figure 4:**
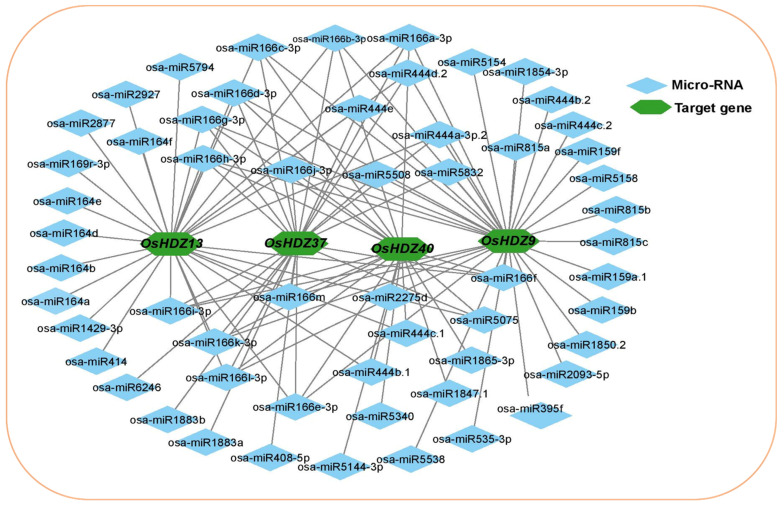
Targeted miRNA sites of the OsHDZIP subfamily III genes in rice represents the functional network assembly of the *HDZIP* genes in *Oryza sativa*. The subfamily III (i.e., *OsHDZIP9, OsHDZIP13, OsHDZIP37,* and *OsHDZIP40*) were mapped to the co-expression database. This analysis revealed 56 unique miRNAs that exhibited various physical/functional interactions, and a network was then assembled based on these interactions.

MicroRNAs are a class of small noncoding regulatory RNAs that control gene expression by directing target mRNA cleavage or translational repression [43]. In recent decades, several investigations have reported that the miRNAs regulate numerous stresses, plant development, and signal transduction. Therefore, to better understand the regulatory mechanism of miRNAs involved in the regulation of *OsHDZIP* genes, 56 putative miRNAs targeting four *OsHDZIP* genes were identified, as shown in the network illustration (Figure 4).

**Figure 5:**
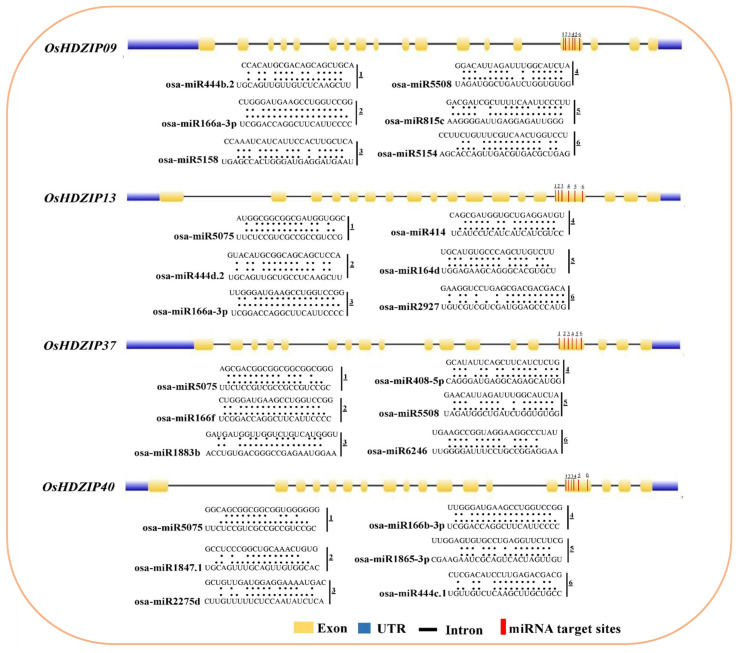
Represents the miRNA target sites on the OsHDZIP subfamily III genes.

The schematic diagrams indicating the *OsHDZIP* genes targeted by miRNAs sites are presented in (Figure 5). Detailed information regarding putative miRNA targeting sites and the *OsHDZIP* genes is provided in the Supplementary Materials (Table S5). The results revealed that among the subfamily III members, *OsHDZIP9* was targeted by 15 miRNAs families (i.e., osa-miR5538, osa-miR535-3p, osa-miR395f, osa-miR2093-5p, osa-miR1850.2, osa-miR159a.1, osa-miR815c, osa-miR815b, osa-miR5158, osa-miR159f, osa-miR444c.2, osa-miR444b.2, osa-miR1854-3p, osa-miR5154, and osa-miR815a). This was followed by *OsHDZIP13*, which was targeted by 11 miRNAs families, including osa-miR414, osa-miR1429-3p, osa-miR164a, osa-miR164b, osa-miR164d, osa-miR164e, osa-miR169r-3p, osa-miR2877, osa-miR2927, osa-miR2794, and osa-miR164f. Furthermore, the same subfamily member, *OsHDZIP37*, was targeted by five miRNAs families (i.e., osa-miR408-5p, osa-miR5832, osa-miR1883a, osa-miR1883b, and osa-miR6246), and *OsHDZIP40* was targeted by only four miRNA families (i.e., osa-miR5144-3p, osa-miR5340, osa-miR444b.1, and osa-miR444c.1). However, from the OsHDZIP subfamily III, four members were targeted in combination by 21 miRNAs families including osa-miR5075, osa-miR1865-3p, osa-miR5075, osa-miR166f, osa-miR2275d, osa-miR444c.1, osa-miR444b.1, osa-miR166e-3p, osa-miR1661-3p, osa-miR166k-3p, osa-miR166i-3p, osa-miRg-33p, osa-miR166h-3p, osa-miR166j-3p, osa-miR444e, osa-miR444d.2, osa-miR444a-3p.2, osa-miR5508, osa-miR166a-3p, osa-miR166b-3p, and osa-miR444d.2. Noticeably, the miRNA165/166 family targeted all the genes in the OsHDZIP subfamily III and could be involved in the post-transcriptional regulation of these genes in rice plants. 

### 3.6. Gene Ontology (GO) Analysis

**Figure 6:**
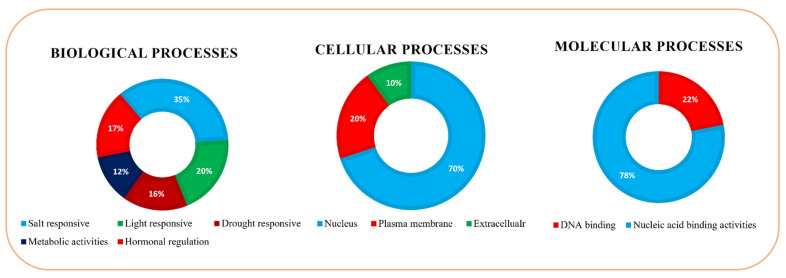
Gene Ontology (GO) enrichment analysis of the *HDZIP* genes in *Oryza sativa*. The data are represented as biological processes, molecular processes, and cellular processes.

The Gene Ontology (GO) enrichment pathway analysis showed various key functions of the *OsHDZIP* genes in *Oryza sativa*. Three functional predictions were analyzed into biological, molecular, and cellular processes [27]. According to the predicted biological processes, *OsHDZIP* genes play a crucial role in growth-related activities via hormonal and metabolic modulation (Figure 6). Additionally, the response to external stimuli and the cellular analysis proved that 36 of the *OsHDZIP* genes were located in the nucleus, 4 genes resided in the plasma membrane, whereas *OsHDZIP28* and *OsHDZIP37* resided in the cytoplasm and may be involved in many cellular-based activities. At the same time, many molecular predictions regarding *OsHDZIP* genes indicated that they are involved in DNA-binding activities. 

### 3.7. Identified cis-Regulatory Elements in OsHDZIP Genes

The *OsHDZIP* in silico analysis revealed that the upstream region of *HDZIP* genes possesses various stress, hormonal, and growth receptive *c*is-regulatory elements. Here, we identified twenty-two cis-regulatory elements. Among these 22 *c*is-elements, 13 were related to stress and growth changes, and 9 were responsive to hormonal changes (Table S2). Further, the hormonal responsive *c*is-regulatory elements, such as AuxRR-Core (auxin-responsive), the GCTCZ-motif (MeJA responsive), and the TCA-salicylic acid element, were responsive. Moreover, ABRE (abscisic acid responsive) was found in most of the *OsHDZIP* genes; meanwhile, the GARE-motif and P-box (a gibberellin-responsive cis-element) were identified in several gene’s upstream regions. Following a drought, the anaerobic induction-responsive *c*is-elements, MYB and ARE, and the light-responsive cis-elements, such as the ZTCT-motif, G-box, and ACE, were also found in the majority of *OsHDZIP* genes. In addition, various stress- and growth-responsive *c*is-elements, such as CAT-box (involved in meristem expression), TC-rich repeats (stress- and defense-responsive *c*is-regulatory elements), MBS1 (regulates flavonoid biosynthesis gene expression), and LTR (low-temperature responsive), were observed in the promoter region of *OsHDZIP* genes. These clustering *c*is-regulatory elements in the promoter region of *OsHDZIP* genes imply their role in regulating gene expression during different growth stages and under environmental stimuli; the same results were reported in [8].

### 3.8. Gene Structure, and Motif Patterns of OsHDZIP Genes

**Figure 7:**
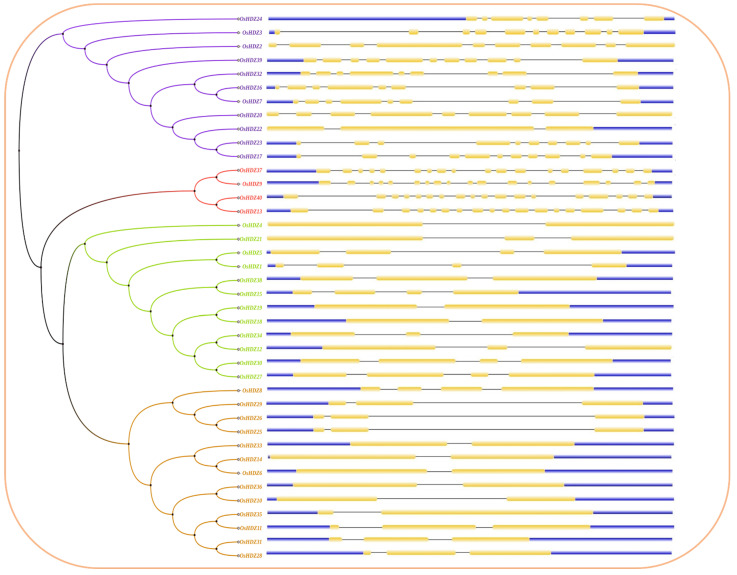
Schematic representation of the HDZIP gene structures in *Oryza sativa*.

We identified the exons–introns distribution using the CDS and genomic sequence of *OsHDZIP* genes. The *OsHDZIP* family genes contain multiple exons and have varied intron lengths (Figure 7). Among these, the *OsHDZIP* subfamily IV and subgroup III were observed with the highest number of intron and exon distribution in which all members (i.e., *OsHDZIP9*, *OsHDZIP13*, *OsHDZIP37*, and *OsHDZIP40*) had 20 exons and 17 introns; followed by subfamily IV in which the *OsHDZIP3* and *OsHDZIP17* had 13 exons and 10 introns; *OsHDZIP16, OsHDZIP20, OsHDZIP32,* and *OsHDZIP39* had 11 exons and 8 introns in total. However, *OsHDZIP2* had 10 exons and 9 introns, and with *OsHDZIP24,* 10 exons and 7 introns were observed. Furthermore, the third-highest number of exons and introns was recorded in subfamily II, in which the *OsHDZIP1, OsHDZIP15, OsHDZIP25, OsHDZIP27,* and *OsHDZIP30* had six exons and three introns, followed by *OsHDZIP34* and *OsHDZIP38* with five exons and two introns; meanwhile, *OsHDZIP18*, *OsHDZIP19*, and *OsHDZIP30* had four exons and two introns; *OsHDZIP21* had three exons and two introns; only *OsHDZIP4* had the least number with two exons and a single intron. Additionally, the OsHDZIP subfamily I was counted as having the least number of introns and exons distributed in which *OsHDZIP11, OsHDZIP25, OsHDZIP26, OsHDZIP28, OsHDZIP29*, and *OsHDZIP31* accounted for five exons and two introns; followed by *OsHDZIP6, OsHDZIP10, OsHDZIP14, OsHDZIP33, OsHDZIP35*, and *OsHDZIP36* with four exons and a single intron; a single gene *OsHDZIP8* was observed with six exons and three introns. 

**Figure 8:**
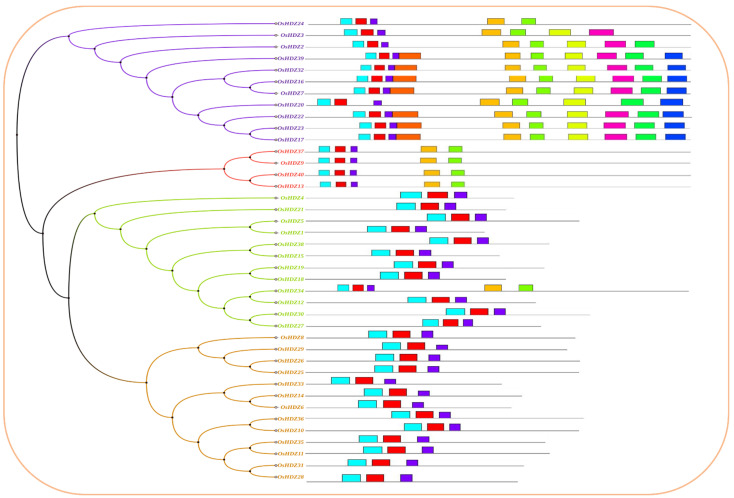
Schematic representation of the conserved motifs of HDZIP genes in *Oryza sativa*.

A total of 40 conserved motifs were discovered using the MEME online server (Version 5.4.1) (accessed on 10 April 2022) [44], and they were found to be appropriate for explaining the *HDZIPs* gene’s structure (Figure 8). Among the 40 *OsHDZIP* genes, the *OsHDZIP7, OsHDZIP8, OsHDZIP9, OsHDZIP16, OsHDZIP17, OsHDZIP22, OsHDZIP23,* and *OsHDZIP32* were counted as having the highest number with 10 motifs; followed by *OsHDZIP2* with 8 motifs and *OsHDZIP3* with 7 motifs. Furthermore, *OsHDZIP9, OsHDZIP13, OsHDZIP24, OsHDZIP34, OsHDZIP37,* and *OsHDZIP40* had five motifs. However, the *OsHDZIP1, OsHDZIP4, OsHDZIP5, OsHDZIP12, OsHDZIP15, OsHDZIP18, OsHDZIP19, OsHDZIP21, OsHDZIP27, OsHDZIP30*, and *OsHDZIP38* in addition to all subfamily I members (i.e., *OsHDZIP6, OsHDZIP8, OsHDZIP10, OsHDZIP11, OsHDZIP14, OsHDZIP25, OsHDZIP26, OsHDZIP28, OsHDZIP29, OsHDZIP31, OsHDZIP33, OsHDZIP35,* and *OsHDZIP36*) counted has having three motifs in total. 

### 3.9. Microarray Expression Analysis of OsHDZIP Genes in Rice Tissues in Developmental Stages under Abiotic/Biotic Stresses and Hormonal Applications 

#### 3.9.1. Microarray Expression Analysis of HDZIP Genes in Developmental Stages

**Figure 9:**
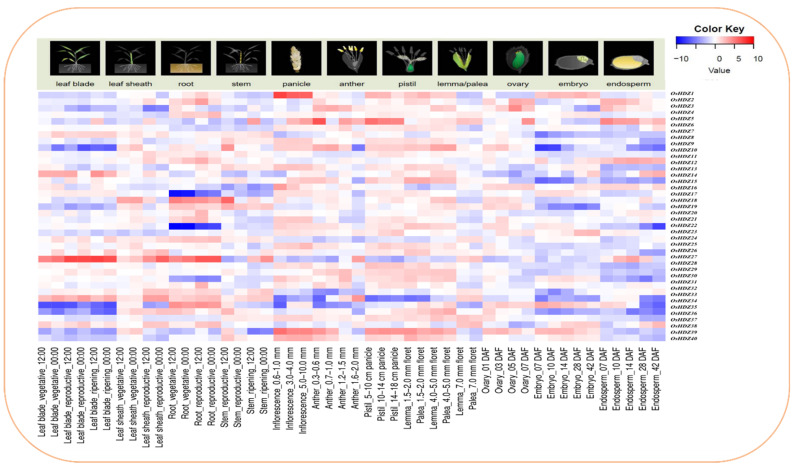
Heatmap of *OsHDZIP* genes’ representation in developmental stages, such as callus, seedlings, leaves at 20 days, shoots, pistil, panicles, endosperm 25 days after pollination (DAP), seed after 5 DAP, pre-emergence inflorescence (Pro-EI), and post-emergence inflorescence (Post-EI) in various tissues. The log2 transformation method normalized and converted the RPKM values displayed.

We examined the different developmental stages and tissue-specific expressions to study the biological roles of *OsHDZIP* genes in plant growth and development based on a set of microarray data obtained from the RiceXPro expression database (version 3.0) (accessed on 13 April 2022) [45]. The microarray expression data analysis of the rice *OsHDZIP* genes family is presented as a heatmap, with blue to red colors reflecting the expression pattern. In twelve tissues (i.e., leaf blade, leaf sheath, roots, stem, inflorescences, anther, pistil, lama, plea, ovary, embryo, and endosperm), the *OsHDZIP* gene family members showed moderate to high expressions, respectively (Figure 9). Among the twelve tissues, *OsHDZIP27* showed dominant expression in leaf blade, leaf sheath, and roots, followed by *OsHDZIP18,* which showed high transcription in roots. Furthermore, *OsHDZIP1, OsHDZIP3, OsHDZIP5, OsHDZIP13*, *OsHDZIP33, OsHDZIP34, OsHDZIP35, OsHDZIP39,* and *OsHDZIP40* showed moderate expression in leaf blade, root, inflorescences, and endosperms. All developmental stages were observed with no or extremely low transcripts, particularly in embryos and endosperm. On the contrary, the leaf blade, root, inflorescence, and anther had high transcription levels. Additionally, the developmental stages, including pistil, lama, and plea, revealed the response of many *OsHDZIP* gene expressions. This expression analysis revealed the essential role of the *OsHDZIP* gene family in developmental stages. 

#### 3.9.2. Expression Analysis of OsHDZIP Genes under Salinity

**Figure 10:**
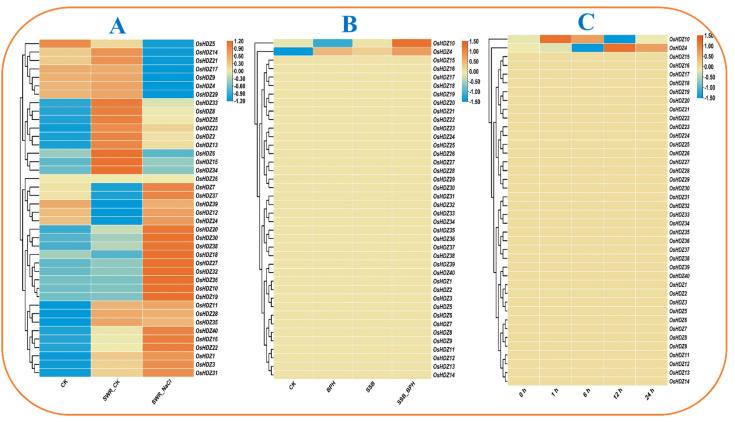
Heatmaps under salinity and biotic stresses: Heatmap (**A**) represents *OsHDZIP* genes’ expression under CK, SWR_CK, and SWR_NaCl; Heatmap (**B**) represents *OsHDZIP* gene expressions in shoot tissues under BPH, SSB, and SSB_BPH; Heatmap (**C**) represents the response of *Cnaphalocrocis medinalis* to stress at different time points in *Oryza sativa*. The log2 transformation method normalized and converted the RPKM values displayed.

Salinity is an important stress that hinders plant growth and yield. The injurious effects of salinity can be noted over the whole plant. To obtain insightful knowledge regarding *HDZIP* genes’ responsiveness to high salinity, the transcriptomic expression data were obtained from the publicly available in the NCBI (GSE102152) (accessed on 17 April 2022) [46]. In the heatmap, the dark orange color on the scale bar represents high expression, whereas the light orange color is moderate, and the blue color genes have no expressions (Figure 10A). Among 40 *OsHDZIP* genes, the dominant transcription was rescored under SWR-NaCl of *OsHDZIP7, OsHDZIP10, OsHDZIP16, OsHDZIP18, OsHDZIP19, OsHDZIP20, OsHDZIP22, OsHDZIP27, OsHDZIP30, OsHDZIP32, OsHDZIP36 OsHDZIP37, OsHDZIP38,* and *OsHDZIP40.* In addition, *OsHDZIP1, OsHDZIP3, OsHDZIP11, OsHDZIP12, OsHDZIP24, OsHDZIP28, OsHDZIP31, OsHDZIP35,* and *OsHDZIP39* were found to have moderate expression, and the rest of the genes did not show a response. Meanwhile, under SWR-CK, various *OsHDZIP* genes (i.e., *OsHDZIP2, OsHDZIP6, OsHDZIP8, OsHDZIP13, OsHDZIP14, OsHDZIP15, OsHDZIP21, OsHDZIP23, OsHDZIP25, OsHDZIP33,* and *OsHDZIP34*) had high expression and moderate expression (i.e., *OsHDZIP1, OsHDZIP3, OsHDZIP4, OsHDZIP8, OsHDZIP9, OsHDZIP11, OsHDZIP29, OsHDZIP31,* and *OsHDZIP35)* in comparison to CK. This expression analysis suggests that *OsHDZIP* genes play a crucial role in the rice plant’s defense against high salinity. 

#### 3.9.3. Expression Analysis of *OsHDZIP* Genes under BPH, SSB, and SSB_BPH

The transcriptomic expression data were obtained from the NCBI (GSE167872) (accessed on 11 May 2022) [47]. The analysis provided insightful predictions of *OsHDZIP* genes involved in rice plant defense against brown planthopper (BPH), rice striped stem borer (SSB), *Chilo suppressalis*, and combined SSB_BPH stresses (Figure 10B). However, in response to BPH, SSB, and the combined stress of SSB_BPH, only two genes, *OsHDZIP4* and *OsHDZIP10,* had dominant expressions. The expression analysis revealed the role of the *OsHDZIP* gene family in plants’ defense against pest infestations. 

#### 3.9.4. Expression Analysis of OsHDZIP Genes under *Cnaphalocrocis medinalis*

The transcriptomic expression data were taken from the NCBI (GSE159259) (accessed on 15 May 2022) [48]. The analysis provided insightful predictions of the *OsHDZIP* gene’s role in rice plant defense against rice leaf folder (RLF) *Cnaphalocrocis medinalis* Guenée (Lepidoptera: Crambidae) (Figure 10C). Further, the response of the *HDZIP* gene family was moderate; among the 40 *OsHDZIP* genes, only *OsHDZIP4* and *OsHDZIP10* were highly expressed at all time points (i.e., 0, 6, 12, and 24 h), and the rest of the genes did not show transcription. This expression analysis suggests that the *HDZIP* gene family plays a role in the rice defense system against biotic stress.

#### 3.9.5. Expression Analysis of OsHDZIP Genes under Brassinosteroids and Jasmonic Acid

**Figure 11:**
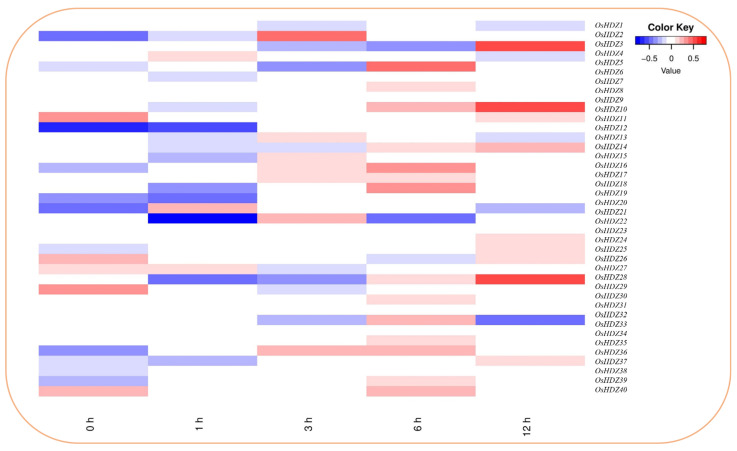
Heatmap of *OsHDZIP* genes’ representation under jasmonic acid; the log2 transformation method normalized and converted the RPKM values displayed.

The expression data of the *OsHDZIP* genes family under Jasmonic acid (JA) and brassinosteroids (BRs) were obtained from the RiceXPro expression database (version 3.0) (accessed on 25 May 2022) [45]. The microarray expression data analysis of the rice *HDZIP* genes family is presented as a heatmap, with blue to red colors reflecting the expression percentage. Under JA, the expression analysis of *OsHDZIP* genes at the 12 h timepoint, *OsHDZIP3, OsHDZIP10* and *OsHDZIP28* and *OsHDZIP11, OsHDZIP24, OsHDZIP25, OsHDZIP26,* and *OsHDZIP37* were found with high and moderate transcription, respectively (Figure 11). Followed by the 6 h timepoint in which only *OsHDZIP5* had high and *OsHDZIP7, OsHDZIP9, OsHDZIP16, OsHDZIP19, OsHDZIP33, OsHDZIP37,* and *OsHDZIP40* had moderate mRNA levels. However, in the 3, 1, and 0 h, *OsHDZIP2* had high, and *OsHDZIP10* and *OsHDZIP11* were observed to have moderate expressions. 

**Figure 12:**
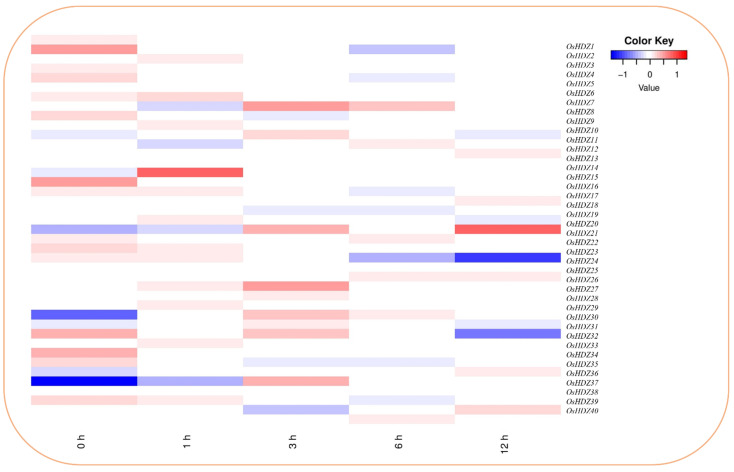
Heatmap of *OsHDZIP* genes’ representation under brassinosteroids; the log2 transformation method normalized and converted the RPKM values displayed.

Under BRs, the *OsHDZIP* genes showed moderate expressions at almost all time points, except *OsHDZIP15* and *OsHDZIP20,* which had high dominant expressions (Figure 12). Further, *OsHDZIP1, OsHDZIP2, OsHDZIP15*, and *OsHDZIP32* (0 h); *OsHDZIP7* (1 h); *OsHDZIP7, OsHDZIP10, OsHDZIP21, OsHDZIP27, OsHDZIP30, OsHDZIP32*, and *OsHDZIP36* (3 h); *OsHDZIP7, OsHDZIP12, OsHDZIP22, OsHDZIP23,* and *OsHDZIP39* (12 h); *OsHDZIP12, OsHDZIP18, OsHDZIP26, OsHDZIP36*, and *OsHDZIP39* (24 h) were found with moderate expressions. However, these findings suggest that *OsHDZIP14* and *OsHDZIP15* showed no response at all given time points. In addition, various genes, such as *OsHDZIP1, OsHDZIP2, OsHDZIP3, OsHDZIP25, OsHDZIP30, OsHDZIP35,* and *OsHDZIP32*, were found with the least transcriptions, and the remaining genes were not expressed. 

### 3.10. Differential Expression of OsHDZIP Genes in Response to Nilaparvata lugens, Laodelphax striatellus Infestations, and JGM Spraying

**Figure 13:**
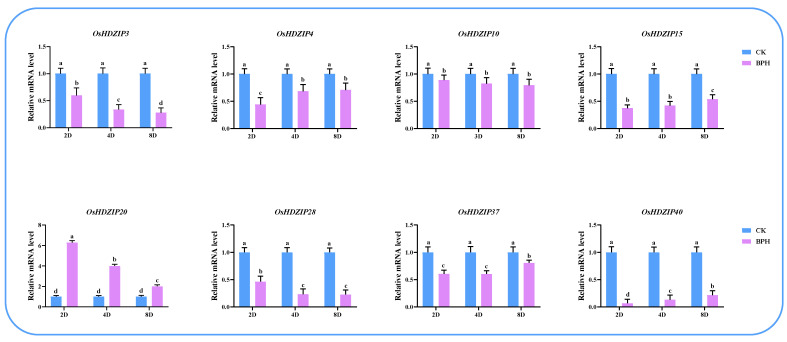
Represents the differential expression analysis of *OsHDZIP* genes (i.e., *OsHDZIP3*, *OsHDZIP4*, *OsHDZIP10*, *OsHDZIP15*, *OsHDZIP20*, *OsHDZIP28*, *OsHDZIP37*, and *OsHDZIP40*) under *Nilaparvata lugens* infestations in *Oryza sativa*.

**Figure 14:**
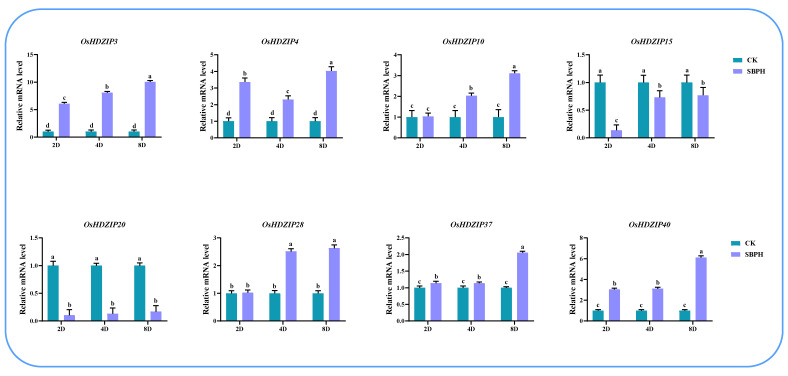
Represents the differential expression analysis of *OsHDZIP* genes (i.e., *OsHDZIP3*, *OsHDZIP4*, *OsHDZIP10*, *OsHDZIP15*, *OsHDZIP20*, *OsHDZIP28*, *OsHDZIP37*, and *OsHDZIP40*) under *Laodelphax striatellus* infestation in *Oryza sativa*.

**Figure 15:**
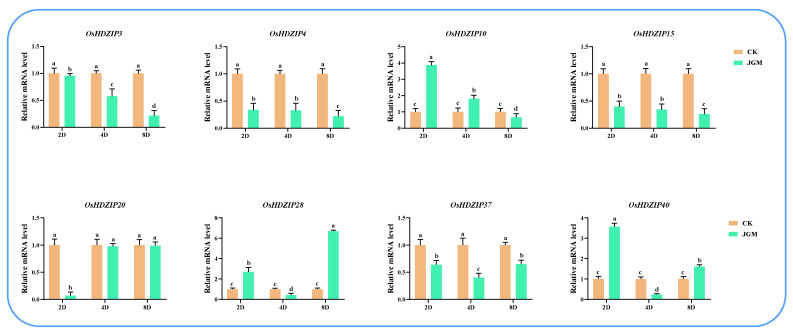
Represents the differential expression analysis of *OsHDZIP* genes (i.e., *OsHDZIP3*, *OsHDZIP4*, *OsHDZIP10*, *OsHDZIP15*, *OsHDZIP20*, *OsHDZIP28*, *OsHDZIP37*, and *OsHDZIP40*) under JGM spraying treatment in *Oryza sativa*.

To further investigate the response of *OsHDZIP* genes under BPH and SBPH infestations and botanical fungicide JGM applications, herein, we performed quantitative real-time polymerase chain reaction (qRT-PCR) to analyze the expression patterns at three-time points over 2 days (2D), 4D, and 8D long treatments during BPH and SBPH infestations and JGM spraying (Figure 13, Figure 14 and Figure 15). The results revealed that the response of ten candidate genes were expressed under all stress conditions.

The BPH is a severe rice pest and causes a huge loss in annual rice production. The expression analysis revealed the important aspect of the *O. sativa* HDZIP transcription factor through moderate and high expressions. Among the eight candidate genes (two genes from each subfamily) at three different time points (Figure 13), moderate to high expression patterns were found, particularly for *OsHDZIP20,* which had the highest expression of six-fold over 2 days of infestation, followed by 4 days with four-fold expression and two-fold after 8 days of BPH infestation. However, *OsHDZIP03, OsHDZIP28*, and *OsHDZIP40* were found with low transcription; in addition, *OsHDZIP04, OsHDZIP10, OsHDZIP15*, and *OsHDZIP37* were found with moderate expressions.

The SBPH is the second most important pest of rice after BPH, which causes a drastic loss to rice plants and their production. To validate the *OsHDZIP* transcription factor response through qRT-PCR under SBPH infestation, we used eight candidate genes at three time points (Figure 14). Among them, *OsHDZIP03* displayed moderate expressions at 2 and 4 days of treatment and had the highest expression at 8 days of infestation up to 10-fold. This was followed by *OsHDZIP40*, with a six-fold expression at eight days. Furthermore, *OsHDZIP04, OsHDZIP10, OsHDZIP15, OsHDZIP28,* and *OsHDZIP37* were found to have moderate expressions; however, *OsHDZIP20* was found to have the lowest expression pattern. 

JGM is a synthetic antibiotic applied to treat rice sheath blight disease, and it is also reported to enhance BPH fecundity; herein, we performed qRT-PCR analysis to validate the *OsHDZIP* gene family’s response under JGM treatment (Figure 15). The obtained results revealed that *OsHDZIP* genes, such as *OsHDZIP10, OsHDZIP20,* and *OsHDZIP40*, present upregulated expressions, whereas *OsHDZIP04, OsHDZIP15*, and *OsHDZIP37* had moderate expressions and a single gene, and *OsHDZIP03, OsHDZIP04*, and *OsHDZIP10* had negligible expression patterns. These results suggest that the *OsHDZIP* participated in rice immunity regulations against JGM spraying applications. 

## 4. Discussion

Plants counter various biotic and abiotic stresses during their life cycle, impairing their biochemical and physiological processes. The plant develops its mechanism to tackle these adverse conditions, such as activation of stress-related TFs and intensified metabolic activities. HDZIP TFs are distributed widely across the plant kingdom and have been recognized for their role in the growth and developmental activities and response to environmental stimuli [7,8,49].

### 4.1. OsHDZIP Genes Are Widely Distributed in the Rice Genome

Phylogenetic trees represent an established method for determining evolutionary changes and functional relationships [50]. The HDZIP protein family has been identified in many species, from mosses to higher plants, such as *Ceratopteris richardii* [51] and *Physcomitrella patens* [52], angiosperms, and gymnosperms [53]. In our study, we conducted a genome-wide survey to determine the phylogenetic relationships and investigate their potential role via qRT-PCR expression analysis; there was a total of 127 HDZIP proteins from rice (40 *OsHDZIP*s), *Cucumis sativus* (40 *CsHZIP*s) [8], and *Arabidopsis thaliana* (47 *AtHDZIP*s) [40]. These proteins were further divided into four subfamilies (Figure 2). The obtained results aligned with previously reported studies on *Arabidopsis thaliana* and *Cucumis sativus* [8,40]. The HDZIP III subfamily was more highly conserved than the other subfamilies. On the other hand, HDZIP I, HDZIP II, and HDZIP IV varied in the numbers of different species [54,55,56]. The conserved number of motifs in *OsHDZIP* genes was analyzed, and the results stated that the HDZIP III and HDZIP IV subfamilies exhibited the highest number of motifs of the other two subfamilies (Figure 8). A motif’s position in each subfamily was highly conserved, supporting their evolutionary classification into different subfamilies [40,55,57].

### 4.2. OsHDZIP Genes Have Tissue Specificity and Play an Integral Role in the Development of Oryza sativa

The HDZIP I TFs were documented for their involvement in plant developmental processes such as root growth and stem elongation, leaf morphology, flowering induction, and pollen hydration [8]. The *OsHDZIP* gene family members, including *OsHDZIP18* and *OsHDZIP27,* displayed dominant expression in all tested organs of *O. sativa* in the developmental stages. Similar results were reported for the *OsHDZIP18*, *OsHDZIP27* homologs *CsHDZ02*, and *CsHDZ33,* respectively. These results indicate the potential importance of *OsHDZIP18*, *OsHDZIP27,* and other subfamily members in regulating the growth and developmental activities of *O. sativa* [8]. 

Furthermore, the subfamily HDZIP II controls hypocotyl elongation, regulates the development of cotyledon and leaves, and involves flower induction [5,8,58]. Here, the *OsHDZIP17* and *OsHDZIP28* were expressed in all six tissues (i.e., root, stem, leaf, panicle, pistil, and embryo) (Figure 9). 

Members of the HDZIP III subfamily were shown to be important for appropriate morphogenesis, embryo development, giving support during the commencement of lateral organ development, control of the shoot apical meristem, and water and nutrient transport throughout the plant’s overall body [6,8,59,60]. Similarly, all of the genes in the HDZIP III subfamily showed transcription in practically all of the investigated tissues (Figure 9), suggesting that they may be important in sustaining rice’s normal growth activities.

Members of the HDZIP IV subfamily have been implicated in developing root hairs, trichome production, anthocyanin biosynthesis, and flowering regulation in plants. [49,61]. *OsHDZIP11* and *OsHDZIP37* were expressed in all the tissues (Figure 9), suggesting that subfamily HDZIP IV plays a potential role in the developmental activity regulations of *O. sativa*.

### 4.3. OsHDZIP Genes Regulate Plant Response to Chewing Insects and other Abiotic Stresses

The homeodomain-leucine zipper transcription factors play a potential role in a plant’s growth and development and are highly responsive to the pronounced effects of stresses including drought [62], cold [63], salinity [64], heat stress [65], heavy metal [66], flooding stress [67], and nutrient stress (iron deficiency) [68]. 

The BPH and SBPH are serious rice pests inflicting damage on a massive scale across Asia [19,21]. Their nymphs and adults cause direct damage by feeding on phloem sap from the tillering to the milking stages of rice and, in the process, transmitting viral pathogens, such as rice ragged stunt virus (RRSV) and rice grassy stunt virus (RGSV) [69,70]. Several studies have identified the potential role of the HDZIP gene family. Until now, no comprehensive study has been conducted to unfold the response of *OsHDZIP* genes to BPH and SBPH infestations. Herein, we performed qRT-PCR on eight candidate genes (two from each subfamily) to validate their expressions. The results revealed the potential role of *OsHDZIP* genes. Furthermore, among these eight candidate genes, *OsHDZIP20* was observed with dominant expressions, and the rest of the genes (i.e., *OsHDZIP04, OsHDZIP10, OsHDZIP15,* and *OsHDZIP37*) had moderate expressions, whereas *OsHDZIP03, OsHDZIP28,* and *OsHDZIP40* had low transcription (Figure 13 and Figure 14). Collectively, the response of these genes uncovered the crucial role of *OsHDZIPs* in rice plant immunity in response to insect pest infestations. However, a functional study is required to investigate the underlying mechanisms inducing the HDZIP gene family response against insect pests. 

Numerous studies highlighted the involvement of HDZIP I and HDZIP II in regulating plant response to abiotic stresses, in particular salinity stress [68]. In our study, *OsHDZIP40, OsHDZIP16, OsHDZIP22, OsHDZIP1, OsHDZIP3, OsHDZIP31, OsHDZIP19, OsHDZIP10, OsHDZIP36, OsHDZIP32, OsHDZIP27, OsHDZIP18, OsHDZIP38, OsHDZIP30, and OsHDZIP20* possessed the dominant expression under combined SWR-NaCl (Figure 10A). Similar expression patterns were observed under SWR-Ck for the *OsHDZIP34, OsHDZIP15, OsHDZIP6, OsHDZIP13, OsHDZIP2, OsHDZIP23, OsHDZIP25, OsHDZIP8,* and *OsHDZIP33* genes in contrast with CK. Collectively, these analyses suggest the responsive role of the HDZIP gene family; further study is required to investigate the role of the HDZIP gene under salinity and chewing and sucking insect combined stress.

The antibiotic JGM was developed in recent decades in China, and it is usually applied two to three times in rice fields to treat rice sheath blight disease (*Rhizoctonia solani*) and fungal infections [25]. Moreover, JGM is reported to be an enhanced controlling agent of the sheath blight disease; however, JGM also has consequences because of its potential role in inducing BPH fecundity [25]. For instance, JGM was applied to the rice plants at the rate of 200 parts per million (ppm), and the results revealed that JGM increased the rice’s resistance to rice sheath blight disease by disrupting the fungal cell wall and reduced sporulation [25]. In addition, JGM was also reported to enhance BPH fecundity [25]. In the current study, the *OsHDZIP* gene showed a differential expression pattern where the candidate genes from each subfamily, including *OsHDZIP40, OsHDZIP28*, and *OsHDZIP10,* were dominantly expressed, whereas the *OsHDZIP3, OsHDZIP4, OsHDZIP15*, and *OsHDZIP37* were moderately expressed (Figure 15). We speculate that these *OsHDZIP* genes are essential for inducing plant immunity to fungal pathogens in rice plants. Further studies are required to elucidate the underlying mechanisms of *OsHDZIP* genes boosting rice immune responses under various insect pest infestations. 

### 4.4. MicroRNAs in Plant–Insect Interaction and Insect Pest Control

Recently, different miRNAs have been identified in numerous species, including *Brassica napus* [71], maize (*Zea mays*) [72], cowpea (*Vigna unguiculata*) [73], soybean (*Glycine max*) [74], pathogen infection in *Arachis hypogaea* [75], and rice [76], and are involved in different metabolism, development, and environmental stresses. In the present study, fifty-six miRNAs belonging to twenty-eight different families were identified and targeted four *OsHDZIP* genes (Table S1). 

The osa-miR166 family targeted four *OsHDZIP* genes, and miR166 has been reported to be involved in drought stress in maize [72], cowpea [73], soybean (Glycine max) seed development [74], peanut disease-resistance [75], and plant growth, development, and stress response in apple [77]. miR444 has been reported to be involved in the cadmium stress regulation of rice [78]. Previously, Anca et al. (2012) reported that the expression profiles of three miRNAs (i.e., osa-miR414, osa-miR408, and osa-miR164) targeting the *OsABP*, *OsDBH* and *OsDSHCT* genes in rice enhanced the miRNAs response against salinity stress [79]. Our results found that four *OsHDZIP* genes (i.e., *OsHDZIP9*, *OsHDZIP13, OsHDZIP37,* and *OsHDZIP40*) were targeted by these miRNAs providing another aspect of these miRNAs’ potential role in regulating rice response under salinity (Figure 4) and their target sites (Figure 5). Collectively, these findings suggest that miRNAs may play vital roles in numerous growth and developmental processes and stress regulation processes by altering the transcriptional level of *HDZIP* genes in *O. sativa*. Among these 28 miRNAs families targeting the *HDZIP* gene family, several miRNAs predicted expression levels and functions have been identified, whereas various miRNAs targeting *OsHDZIP* genes that regulate biotic/abiotic stress responses and other important agronomic traits remain to be clarified.

### 4.5. Expression Analysis of HDZIP Gene Family under Hormonal Applications

JA is synthesized from linolenic acid through the action of several enzymes in plant chloroplast membranes, and the current evidence indicates that it induces resistance against necrotrophic pathogens and chewing herbivores [80,81]. In comparison, the microarray data showed that the expression of *OsHDZP3, OsHDZP9*, and *OsHDZP28* was enhanced, suggesting their possible involvement in JA-mediated rice resistance against BPH (Figure 11). 

BRs are a group of polyhydroxylated steroidal phytohormones that are mandatory for plant development, growth, and productivity [82]. In addition to their significant involvement in growth-related activities, BRs are a key stress hormone [27]. Herein, the upregulated expressions of *OsHDZIP15* and *OsHDZIP20* suggest the essential role of these genes and their possible participation in the immunity regulation of the rice plant; however, functional studies are required to unfold the underlying mechanism of these crucial hormones (Figure 12).

## 5. Conclusions

Forty *OsHDZIP* family TFs were identified in the rice genome database and classified into four subfamilies based on their domain and structural properties. Furthermore, heatmap analysis revealed the expression of *OsHDZIP* TFs in distinct *O. sativa* tissues. The expression of *OsHDZIP* genes under salinity and hormone stress indicated that they might play a role in modulating *O. sativa* resistance. Furthermore, differential expression patterns were found under JA and BR treatments, indicating that *OsHDZIP* genes may be critical in hormonal-mediated rice immunity against BPH and SBPH. On the other hand, stress control is a complicated system, and our results suggested the potential application of HDZIP biomarkers in developing stress-resilient rice lines. These results provide the missing role of *HDZIP* genes in regulating rice immunity against SBPH and particularly BPH. 

## Figures and Tables

**Table 1:** The gene and protein features of *HDZIP* genes family members in *Oryza sativa*.

Name	Locus ID	Subgroups	Chr	Start	End	AA	Mw (kDa)	PI	SL
*OsHDZ1*	LOC_Os01g45570	II	1	25,883,718	25,880,204	229	229	9.00	N
*OsHDZ2*	LOC_Os01g55549	IV	1	32,009,784	32,006,374	816	88,638.79	6.43	N
*OsHDZ3*	LOC_Os01g57890	IV	1	33,471,900	33,478,535	709	78,179.26	6.97	P
*OsHDZ4*	LOC_Os02g05640	II	2	2,757,693	2,758,714	237	25,593.73	9.34	N
*OsHDZ5*	LOC_Os02g35770	II	2	21,489,894	21,487,595	349	37,437.65	5.65	N
*OsHDZ6*	LOC_Os02g43330	I	2	26,143,309	26,144,731	261	28,537.38	4.84	N
*OsHDZ7*	LOC_Os02g45250	IV	2	27,494,914	27,487,865	804	86,047.24	5.55	P
*OsHDZ8*	LOC_Os02g49700	I	2	30,381,303	30,383,661	343	37,614.24	4.70	N
*OsHDZ9*	LOC_Os03g01890	III	3	549,823	557,909	839	91,828.58	5.74	N
*OsHDZ10*	LOC_Os03g07450	I	3	3,786,933	3,784,283	366	39,623.72	6.40	N
*OsHDZ11*	LOC_Os03g08960	I	3	4,652,927	4,654,824	311	33,612.78	4.68	N
*OsHDZ12*	LOC_Os03g12860	II	3	6,931,709	6,933,328	292	30,516.17	8.64	N
*OsHDZ13*	LOC_Os03g43930	III	3	24,657,650	24,651,800	862	93,747.56	6.19	P
*OsHDZ14*	LOC_Os04g45810	I	4	27,124,166	27,125,512	276	30,653.13	5.19	N
*OsHDZ15*	LOC_Os04g46350	II	4	27,479,717	27,477,579	247	27,291.10	9.06	N
*OsHDZ16*	LOC_Os04g48070	IV	4	28,607,038	28,600,698	813	86,758.01	5.59	N
*OsHDZ17*	LOC_Os04g53540	IV	4	31,907,122	31,899,718	784	85,259.35	5.59	N
*OsHDZ18*	LOC_Os06g04850	II	6	2,123,398	2,124,787	256	27,016.66	9.12	N
*OsHDZ19*	LOC_Os06g04870	II	6	2,137,450	2,139,101	308	32,084.86	9.14	N
*OsHDZ20*	LOC_Os06g10600	IV	6	5,502,411	5,499,342	697	75,491.69	6.19	N
*OsHDZ21*	LOC_Os06g48290	II	6	29,199,356	29,198,270	256	27,590.97	9.13	N
*OsHDZ22*	LOC_Os08g04190	IV	8	2,037,504	2,034,454	749	80,854.03	5.97	N
*OsHDZ23*	LOC_Os08g08820	IV	8	5,109,238	5,116,781	784	84,615.36	5.50	N
*OsHDZ24*	LOC_Os08g19590	IV	8	11,702,603	11,711,044	786	86,649.10	8.34	N
*OsHDZ25*	LOC_Os08g32080	I	8	19,888,358	19,884,012	349	37,386.74	4.59	N
*OsHDZ26*	LOC_Os08g32085	I	8	19,888,358	19,884,012	349	37,386.74	4.59	N
*OsHDZ27*	LOC_Os08g36220	II	8	22,831,125	22,829,282	354	36,889.29	7.02	N
*OsHDZ28*	LOC_Os08g37580	I	8	23,805,452	23,803,380	269	28,928.92	4.64	C
*OsHDZ29*	LOC_Os09g21180	I	9	12,783,649	12,780,659	333	35,698.53	5.08	N
*OsHDZ30*	LOC_Os09g27450	II	9	16,676,359	16,674,571	362	37,833.06	6.22	N
*OsHDZ31*	LOC_Os09g29460	I	9	17,903,164	17,905,338	277	29,392.27	4.62	N
*OsHDZ32*	LOC_Os09g35760	IV	9	20,574,897	20,567,982	872	91,720.25	5.75	N
*OsHDZ33*	LOC_Os09g35910	I	9	20,671,754	20,673,282	249	27,300.31	5.62	N
*OsHDZ34*	LOC_Os10g01470	II	10	286,941	284,701	247	27,178.62	8.15	N
*OsHDZ35*	LOC_Os10g23090	I	10	12,041,604	12,043,235	305	32,734.71	5.05	N
*OsHDZ36*	LOC_Os10g26500	I	10	13,804,811	13,806,732	355	36,848.13	6.05	N
*OsHDZ37*	LOC_Os10g33960	III	10	18,085,536	18,092,500	840	92,132.16	5.58	C
*OsHDZ38*	LOC_Os10g41230	II	10	22,152,631	22,154,163	311	33,143.22	9.09	N
*OsHDZ39*	LOC_Os10g42490	IV	10	22,916,203	22,910,460	882	94,667.18	5.63	N
*OsHDZ40*	LOC_Os12g41860	III	12	25,927,353	25,920,639	855	93,109.96	5.97	P

Amino acid: AA; molecular weight: MW; nuclear: N; plasma membrane: P; cytoplasmic: C; isoelectric point: PI; subcellular location: SL.

## Data Availability

Not applicable.

## Supplementary Materials

The following supporting information can be downloaded at: https://www.mdpi.com/article/10.3390/bioengineering9080398/s1, Table S1: Primer sequences for RT-qPCR, Table S2: Types and number of *c*is-acting regulatory elements analysis involved in growth, development, and stress and hormonal response; Table S3: Gene structure and domain distribution; Table S4: Predicted functional partners of *OsHDZIP* proteins; Table S5: A detailed list of miRNAs.
