# Supplementary material for: The Homeodomain-Leucine Zipper Genes Family Regulates the Jinggangmycin Mediated Immune Response of Oryza sativa to Nilaparvata lugens, and Laodelphax striatellus
Source: Bioengineering (Basel). 2022 Aug 17;9(8):398. doi: 10.3390/bioengineering9080398 (PMC9405480; doi:10.3390/bioengineering9080398)
Supplement: Supplementary file 1 [file bioengineering-09-00398-s001.zip › Supplementary Data Table S1, S2, S3, S4 HDZIP Supply Data.pdf]

The Homeodomain-Leucine Zipper genes family regulate the response of *Oryza sativa* to *Nilaparvata lugens*, *Laodelphax striatellus*, and Jinggangmycin

Sheraz Ahmad[1], Yu Chen[1], Amir Zaman Shah[1], Huaiqi Wang[1], Xi Chuanyuan[1], Haowen Zhu[1] and Linquan Ge[1]

1 College of Horticulture and Plant Protection, Yangzhou University, Yangzhou, China.

*Correspondence: lqge@yzu.edu.cn

Table S1: Primer sequences for RT-qPCR.

Genes	**Primer's sequence (5'to3')**
OsHDZ3	*F:* GATCCAATCCATAAGGCCAAAC<br>*R:* CTCCGAAGACGAGTCCATTCATAC
OsHDZ4	*F:* GGTGTTGCTGTTCGGTGAGGTGCT<br>*R:* TCCGCCGCTGCCTCGTCGTCGTA
OsHDZ10	*F:* GCAGCAACCGCAGCGAGAATAGC<br>*R:* AGAACGGGATCATGCCGCCACC
OsHDZ15	*F:* GCAGACGGAGGTGGACTGCGAGTT<br>*R:* GCGCTGGAGTTGTTATTGGTGTTGGA
OsHDZ20	*F:* TGAATGGCATGGGAGGGAGAAGC<br>*R:* GCGGCACATTGTCCATCGCGTAT
OsHDZ28	*F:* TCGTCGGTGAAGGAGGAGGAGGACC<br>*R:* TTGAGAAGCTGCCCGTGGAAGAACA
OsHDZ37	*F:* GCACTACGGCACATCAGACAAA<br>*R:* CACCCATGACAGACCAACCATC
OsHDZ40	*F:* CGACGCAATACTGTGCTGTTCT<br>*R:* AGCGAGATGTCCTGGAGGTTGA
ACTIN	*F:* TCCATCTTGGCATCTCTCAG<br>*R:* GTACCCGCATCAGGCATCTG

Table S2: Types and number of *c*is-acting regulatory elements analysis involved in the growth and development, stress and hormonal response.

	**Site Name**	**Function**
**Hormones**	AuxRR-core	*Cis*-acting regulatory element involved in auxin responsiveness
	CGTCA-motif	*Cis*-acting regulatory element involved in the MeJA responsiveness
	CGTCA-motif	*Cis*-acting regulatory element involved in the MeJA responsiveness
	P-box	gibberellin-responsive element
	TCA-element	*Cis*-acting element involved in salicylic acid responsiveness
	TCA-element	*Cis*-acting element involved in salicylic acid responsiveness
	ABRE	*Cis*-acting element involved in the abscisic acid responsiveness
	GARE-motif	gibberellin-responsive element
	ABRE	*Cis*-acting element involved in the abscisic acid responsiveness

**Stress and growth**	G-Box	*Cis*-acting regulatory element involved in light responsiveness
	G-Box	*Cis*-acting regulatory element involved in light responsiveness
	ATCT-motif	part of a conserved DNA module involved in light responsiveness
	ACE	cis-acting element involved in light responsiveness
	GC-motif	enhancer-like element involved in anoxic specific inducibility
	ARE	*Cis*-acting regulatory element essential for the anaerobic induction
	MBS	MYB binding site involved in drought-inducibility/resistance
	TATA-Box	core promoter element around -30 of transcription start
	CAAT-box	common cis-acting element in promoter and enhancer regions
	LTR	cis-acting element involved in low-temperature responsiveness
	MBS1	MYB binding site involved in flavonoid biosynthetic genes regulation
	TC-rich repeats	cis-acting element involved in defense and stress responsiveness
	CAT-box	cis-acting regulatory element related to meristem expression

Table S3: Domain distribution and structure of *OsHDZIP* genes in *Oryza sativa*.

No.	Motif Logo	Motif Sequence
1	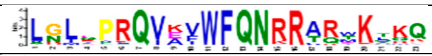	LGLEPRQVKVWFQNRRARWKTKQ
2	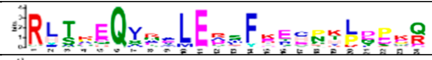	RLTKEQVRALERSFKECPKLDPKQ
3	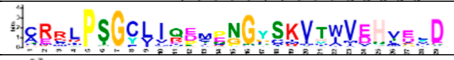	CRRLPSGCLIQEMPNGYSKVTWVEHVELD
4	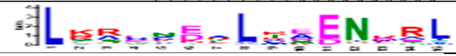	LKRENEALREENRRL
5	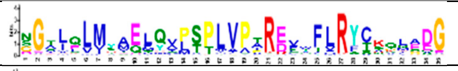	NGTJQLMYAELQVPSPLVPTREVWFLRYCKQLADG
6	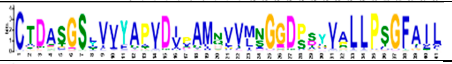	CTDASGSLVVYAPVDIPAMNVVMNGGDPSYVALLPSGFAIL
7	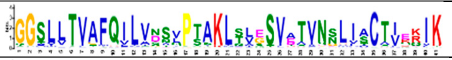	GGSLLTVAFQILVNSVPTAKLSLESVATVNSLIACTIEKIK
8	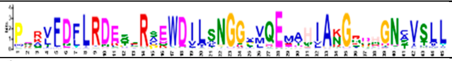	PERVFDFLRDESSRSEWDILSNGGVVQEMAHIAKGRDHGNCVSLL
9	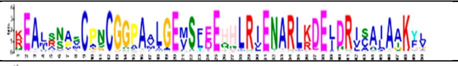	KEALSNASCPNCGGPAALGEMSFEEHHLRIENARLKDEJDRISAJAAKYL
10	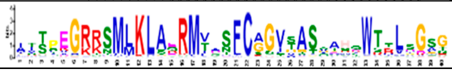	ITTPEGRRSMLKLAERMVASFCAGVSASSAHZWTTLSGSG

Table S4 Predicted functional partners of OsHDZIP proteins

**RoC9**	**Homeobox-leucine zipper protein ROCP; Probable transcription factor; belongs to the HDZIP homeobox family. Class IV subfamily. (816aa)**
**Predicted functional partners:**	
**Q3V826**	Os02g0682500 protein
A0A0N7KLN2	Os02g0183800 protein
OSHAP3E	cDNA clone: 002-134-B07, full insert sequence

Q0JAK5	cDNA clone: J03305D05, full insert sequence
Q7F3D6	cDNA clone: 002-141-A12, full insert sequence
LFL1	B3 domain-containing protein LFL1; Transcription repressor involved in flowering time regulations.
Q8GVN6	Os07g0531600 protein
AP2-5	APETALA2-like protein 5; Transcription factor. Involved in spikelet transition and development.
A0A0P0VDU4	Os02g0114400 protein
A0A0P0WRG0	Os05g0586300 protein
